# The Use of H_2_ in Catalytic Bromate Reduction by Nanoscale Heterogeneous Catalysts

**DOI:** 10.3390/nano12071212

**Published:** 2022-04-04

**Authors:** Nurbek Nurlan, Ainash Akmanova, Woojin Lee

**Affiliations:** 1 Pharmacology and Toxicology, School of Medicine, Nazarbayev University, Nur-Sultan 010000, Kazakhstan; nurbek.nurlan@nu.edu.kz; 2 Green Energy and Environmental Research Group, National Laboratory Astana, Nazarbayev University, Nur-Sultan 010000, Kazakhstan; ainash.akmanova@nu.edu.kz; 3 Civil and Environmental Engineering, School of Engineering and Digital Sciences, Nazarbayev University, Nur-Sultan 010000, Kazakhstan

**Keywords:** hydrogen use, solid-state H_2_, bromate reduction, nanocatalysts

## Abstract

The formation of bromate (BrO_3_^−^)in groundwater treatment is still a severe environmental problem. Catalytic hydrogenation by nanoscale heterogeneous catalysts with gaseous H_2_ or solid-state H_2_ has emerged as a promising approach, which relies on reducing BrO_3_^−^ to innocuous Br^−^ via the process of direct electron transfer or reduction with atomic hydrogen. Several nanocatalysts have demonstrated high efficiency with a 100% effective BrO_3_^−^ reduction with greater than 95% of Br^−^ generation in the batch and continuous reactors. However, this technology has not been widely adopted in water treatment systems. Indeed, this research article summarizes the advantages and disadvantages of these technologies by highlighting the factors of nanomaterials reduction efficiency, long-term durability, and stability, as well as addressing the essential challenges limiting the implementation of the use of H_2_ for BrO_3_^−^ reduction. In this work, we provide an economic evaluation of catalytic BrO_3_^−^ removal, safe hydrogen supply, storage, and transportation.

## 1. Introduction

Bromate (BrO_3_^−^) is the most common disinfection byproduct in drinking water treatment systems, and its contamination has become a significant barrier to its use in the disinfection process. BrO_3_^−^ is a category I and group B2 carcinogen; therefore, its maximum permissible concentration has been set to 10 µg/L by World Health Organization (WHO) [1,2,3]. It is primarily originated from the ozonation process where the bromate precursor, bromide (Br^−^), derived from natural and anthropogenic sources, leads to the formation of hypobromous acid (HBrO) and hypobromite (BrO^−^). These can be subsequently oxidized to bromate by ozone radicals [4]. Due to the well-developed process of sodium hypochlorite manufacturing, BrO^−^ can also be formed from the frequent presence of aqueous bromide in the chlorine disinfection process [5]. In recent years, the catalytic hydrogenation of BrO_3_^−^ reduction by nanoscale heterogeneous catalysts has emerged as a promising solution to the need to control bromate in disinfected groundwater and drinking water. 

Catalytic BrO_3_^−^ reduction technology has a series of distinct advantages. Due to its fast reaction kinetics towards contaminants, high efficiency, and extended durability, bromate reduction by nanoscale heterogeneous catalysts could be potentially applied to current groundwater and surface water treatment systems [6]. Based on the evaluation of significant environmental factors, these technologies exhibited reliable stability and cost-effectiveness [7]. Moreover, the catalysts with potential applications were able to reduce BrO_3_^−^ efficiently using H_2_-releasing chemical agents, while the catalytic bromate reduction has been widely reported in the presence of H_2_ gas [7,8]. This has been considered a significant hurdle to applying the catalyst process to the natural water and groundwater treatment plant due to safety issues [9]. Numerous studies have evaluated and assessed overall the catalytic reduction technologies available; however, a review on the use of H_2_ for the catalytic hydrogenation of BrO_3_^−^ has not been conducted yet. The current prospects of catalytic technology for the successful reduction of BrO_3_^−^ need to be updated in light of recent studies. 

The main objectives of this review are to provide a summary of the relevant reaction mechanisms and implications of catalytic technology with and without H_2_ gas and with H_2_-releasing chemical agents for the enhanced reduction of BrO_3_^−^ in the water and groundwater treatment process focused on the following aspects: (1) nanoscale heterogeneous materials’ catalytic performance for BrO_3_^−^ hydrogenation, (2) the safe and efficient use of H_2_ sources, (3) the effect of H_2_ use on the long-term durability of nanomaterials. An economic evaluation of the technologies is given, followed by a discussion on the safe and efficient use of H_2_. The review paper concludes by suggesting the future research needed for removing bromate and prioritizing H_2_ use in order to achieve full-scale potential application in water and groundwater treatment.

## 2. Reaction Mechanism of Catalytic BrO_3_^−^ Removal 

The reduction of BrO_3_^−^ by nanoscale heterogeneous catalysts is a multi-step redox reaction requiring noble and/or promoter metals in their reduced forms and an additional reducing agent (H_2_ or borohydrides). The primary reaction mechanism of catalytic BrO_3_^−^ reduction can be described through sequential reactions commonly shown in most of the oxyhalide contaminants, i.e., (1) ${\mathrm{BrO}}_{3}^{-}+2H^{+}+2e^{-}\to {\mathrm{BrO}}_{2}^{-}+{\;H}_{2}O$; (2) ${\mathrm{BrO}}_{2}^{-}+2H^{+}+2e^{-}\to {\mathrm{BrO}}^{-}+{\;H}_{2}O$; (3) ${\mathrm{BrO}}^{-}+2H^{+}+2e^{-}\to {\mathrm{Br}}^{-}+{\;H}_{2}O$ [10,11]. Before the initiation of catalytic BrO_3_^−^ hydrogenation, the nanoscale heterogeneous catalysts need to be in a reduced form, i.e., zerovalent metals, to reduce the BrO_3_^−^. Process 1: the reaction is able to start with the adsorption of BrO_3_^−^ on the catalysts’ surface where BrO_3_^−^ is reduced to Br^−^. Hamid et al. reported that the activation of H_2_ to reactive H_ads_ (Pd^0^ + H_2_ → Pd-2H_ads_) plays a vital role in catalytic hydrogenation by nanoscale heterogeneous catalysts [12]. The reactive H_ads_ is responsible not only for the reduction of BrO_3_^−^ but also for the reduction of the metal. Process 2: the adsorbed BrO_3_^−^ on the surface of catalysts is able to be reduced to Br^−^ by direct electron transfer from the active catalytic sites [13]. Process 3: H_2_ gas from an external source or hydrolysis of reductants (e.g., NaBH_4_) further reacts with bromate in the bulk phase of suspension [14]. Hydrogen could also be formed via the water-splitting reaction of nanoscale heterogeneous catalysts in a bulk aqueous solution. 

## 3. Nanoscale Heterogeneous Catalysts for BrO_3_^−^ Reduction 

### 3.1. Direct Bromate Reduction by Nanoscale Heterogeneous Catalysts without H_2_

Several nanocatalysts, e.g., zerovalent metals with different supporting materials, have been extensively studied in order to efficiently reduce BrO_3_^−^ via direct electron transfer without H_2_. Various noble and trace metals such as Pd, Pt, and Ru on an activated carbon support, nanoscale-zero-valent iron on modified activated carbon (NZVI/MAC), and Green Nanoscale-Zero-Valent Iron (G-NZVI) catalysts showed an excellent level of performance in eliminating BrO_3_^−^ when no hydrogen was present in their reactions [15,16]. The G-NZVI and NZVI/MAC catalysts showed an enhanced reduction of BrO_3_^−^ at 2 and 5 min with negligible adsorption on the surface by generating ~100% and ~83% of Br^−^, respectively (Table 1). These catalysts are low-cost materials with high reductive capacity, and their application in water and groundwater treatment has the potential to attract intense interest. At the same time, Pd, Pt, and Ru supported on the activated carbon demonstrated much slower kinetics, with a complete conversion of BrO_3_^−^ to Br^−^. The possible advantages of a nanoscale heterogeneous catalyst system without gaseous H_2_ are a safe and easy operation process, cost-effectiveness, and remarkable efficiency in the removal of bromate. However, the catalyst system might have distinct disadvantages in terms of the catalyst’s degree of durability and sustainability. The catalytic reactivity of Pd/C and Pt/C gradually decreased over multiple cycles of catalyst usage; however, Ru/C showed durability for five consecutive cycles with a moderate loss of reduction activity [13]. Moreover, the present environmental remediation technologies require more stable and reactive catalytic materials for a long-term operation. Therefore, using gaseous H_2_ or alternative H_2_ sources could enhance reactivity and prolong longevity during the successful process of BrO_3_^−^ removal designed to be implemented in actual water and groundwater treatment systems. 

### 3.2. Catalytic Bromate Reduction by Nanoscale Heterogeneous Catalysts with H_2_

To enhance the reactivity of the nanoscale catalysts during the direct reduction of bromate, the H_2_ induced-catalytic approach using various materials has been studied in batch and continuous reaction systems [19,25]. The H_2_, a reducing agent, needs to be provided for the hydrogenation of BrO_3_^−^ and the reduction of metals. Compared to heterogeneous catalysts without an H_2_ source, the primary advantages of those with gaseous H_2_ are the faster reaction kinetics and the complete removal of BrO_3_^−^ to Br^−^. For instance, a successful and efficient BrO_3_^−^ reduction was achieved by Pd/Cu-Y (metals over faujasite zeolite) and mono and bimetallic on Zeolite Socony Mobile-5 (Cu-Pd)-ZSM5 in 2 and 10 min, respectively, while only 70% of BrO_3_^−^ was removed with only H_2_ in 120 min (Table 1) [19,21]. Various metals, including Pd, Pt, Rh, and Ru, were deposited on the surface of activated carbon, also resulting in the complete conversion of BrO_3_^−^ to innocuous Br^−^ within 30 min [18]. 

The presence of gaseous H_2_ facilitated the reduction process by accelerating and simultaneously decreasing the surface passivation of catalysts. It is a well-known fact that the bromate reduction mechanism by metals involves H_2_ chemisorption on the metal surface, and the most active catalysts usually form medium-strength bonds with hydrogen [26]. It has been found that Pd metal adsorbs H_2_ on its surface and subsequently activates it for the process of bromate removal (Pd + H_2_ → 3H_ads_–Pd). Therefore, a continuous, constant supply of H_2_ gas (20 mL/min) prolongs the catalyst’s lifetime, ensuring its long-term durability. When compared to control experiments, the bimetallic catalyst supported by NZVI (Cu/Pd-NZVI) in continuous operation mode showed a sustainable reduction of BrO_3_^−^ for 11 h with >99% removal [12]. At optimized conditions, the complete removal of BrO_3_^−^ to Br^−^ was conserved for 24 h with a slight catalyst surface passivation over the next 100 h (Table 2). In addition, (Pd, Ru)-CNF Carbon Nanofiber/monolith catalysts demonstrated only a 10% loss in reactivity after 7 h of continuous BrO_3_^−^ reduction [17]. The superior catalytic performance and longevity depends primarily on the hydrogen chemisorption ability of metals and nanomaterials. Thus, the higher adsorption capacity of H_2_ led to a higher degree of catalytic activity.

Several studies reported a decrease in the catalytic reactivity of nanoscale heterogeneous catalysts during BrO_3_^−^ reduction without H_2_, which was due to the formation of OH^−^ ions hindering the contact of BrO_3_^−^ on active catalyst sites, while the constant purging with H_2_ resulted in a decline of suspension pH, maintaining their catalytic reactivity [12,15,27]. However, there are certain limitations involved in using gaseous H_2_ from an economic and/or a technical perspective. The limited solubility of gaseous H_2_ could lead to an inevitable waste of resources. An adequate supply of H_2_ is also needed for reducing and regenerating nanocatalysts before BrO_3_^−^ reduction; a significantly high concentration of H_2_ and preparation and equilibration times are required for a successful operation. For example, such as when Pd/Cu-Y and Pd-mesoporous carbon nitride catalysts were reduced for 3 to 4 h under hydrogen flow [20]. Another disadvantage may be that due to the stirring or mechanical shaking during BrO_3_^−^ reduction, the pre-purged H_2_ is still able to escape from the reaction solution.

### 3.3. Catalytic Bromate Reduction by Nanoscale Heterogeneous Catalysts with Solid-State H_2_

Solid-state H_2_ producing chemical compounds have attracted considerable attention in removing water contaminants because they release highly pure H_2_ gas [24,27,28]. The primary advantage of solid H_2_ releasing compounds (e.g., borohydrides) is that they offer a more practical and feasible approach as compared to gaseous H_2_ [23]. The H_2_ produced from the catalytic water and groundwater treatment system with solid-state H_2_ is able to be much more controllable than gaseous H_2_ and is directly and instantly managed. However, borohydrides do not possess effective catalytic reductant properties; therefore, they do not efficiently remove BrO_3_^−^ in water and groundwater because of their slow kinetics of self-hydrolysis. The hydrolysis of borohydrides can be accelerated using heterogeneous catalysts. For instance, the catalytic reduction efficiency of BrO_3_^−^ by the Zeolite-Imidazole Framework (ZIF-67), Material Institute Lavoisier (MIL-88A), as well as 4,4′-bipyridine and 1,3,5-benzenetricarboxylic acid ligands (Ni(4,4′-bipy)(1,3,5-BTC)) in the presence of NaBH_4_ showed an effective reduction in 60 min with >95% formation of Br^−^ (Table 1) [22,23,24]. Furthermore, hydrogen evolution during BrO_3_^−^ reduction is able to be controlled by the concentration of borohydrides and different catalyst loadings. Compared to the process of BrO_3_^−^ removal by nanomaterial catalysts with gaseous H_2_, a more efficient BrO_3_^−^ reduction integrated with solid borohydride hydrolysis is achievable using non-noble metals [29]. The durable and sustainable catalytic reactivity for several cycles of BrO_3_^−^ reduction can be considered another advantage of using solid borohydrides, e.g., ZIF-67 and Ni(4,4′-bipy)(1,3,5-BTC) which continuously showed a complete removal in the 5th and 6th cycles (Table 2) [22,23]. A disadvantage of using solid-state H_2_ chemical compounds is the need for secondary treatment to treat NaBH_4_ byproducts (boron species, i.e., NaBO_2_) formed after their hydrolysis, which often impedes their industrial application [30]. 

## 4. An Economic Evaluation of Catalytic BrO_3_^−^ Reduction

The apparent advantages of the catalytic reduction system with H_2_ in water and groundwater treatment raises the question, “Why has the drinking water industry still not adopted this technology to reduce and remove the inevitable contaminants?” The primary reasons given are related to the cost-and-safety-associated uncertainties involved. Noble metals are expensive, and the amounts required for the process of BrO_3_^−^ reduction depend significantly on their level of catalytic reactivity and durability during the treatment of contaminated water and groundwater. The optimal ranges of these significant factors vary from one study to the other because of the reactor design, catalyst material type, and operating conditions. Moreover, the H_2_ required for BrO_3_^−^ reduction is also costly and potentially dangerous to store and transport [31]. These potential concerns can hinder the implementation of this emerging and promising catalyst technology in potable water and groundwater purification. 

The transportation and storage of gaseous H_2_ for use in the treatment technology raises potential safety concerns [32]. Several H_2_ storage techniques have been proposed to date, including compressed hydrogen, metal hydrides, borohydrides, cryogenic liquid hydrogen, and carbon nanotube and metal–organic framework H_2_ storage systems [33,34,35,36,37]. Among them, compressed H_2_ gas storage appears to be a suitable strategy because of its cost-effectiveness, operation simplicity, and high efficiency that is based on time and technological feasibility factors [38]. One review questioned why the catalytic bromate removal system using H_2_ still has not been implemented in the existing water treatment plants, while compressed H_2_ has already been popularly used in commercial fuel cell vehicles and the environmental catalyst technology for this has been so extensively developed [38,39,40,41]. Compressed H_2_ storage tanks can be used for the molecular hydrogen supply; however, this approach might require proper gas handling technology in the water and groundwater treatment.

To efficiently use hydrogen during the process of BrO_3_^−^ reduction, the continuously supplied H_2_ needs to be adequately consumed within the retention time of the reactor. This indicates that the BrO_3_^−^ consumption rate in the reactor is managed by the H_2_ flow rate, or the hydrogen releasing rate is equivalent to the BrO_3_^−^ interaction time on the active catalyst sites. All the gaseous H_2_ released to the reactor needs to be adsorbed on the nanocatalysts and be further used for the BrO_3_^−^ reduction before leaving as an off-gas. In the case of solid-state H_2_, its amount with the flow rate can be calculated easily with the borohydride dosage and catalyst concentrations [24]. Another approach for obtaining a potential near-future H_2_ supply might be the introduction of water-splitting catalysts and/or their combinations with the nanoscale heterogeneous catalysts in order to simultaneously produce H_2_ during the reduction of BrO_3_^−^ [42,43,44].

The high cost of the hydrogen required for the BrO_3_^−^ reduction is another factor that significantly impacts its implementation in actual water and groundwater treatment. However, to address this concern, less expensive alternative techniques for producing H_2_ need to be investigated that are specifically designed for the treatment, e.g., coal (1.89 $/kg), nuclear-assisted electrolysis (2.24 $/kg), natural gas (3.50 $/kg), geothermal (4.38 $/kg), biomass (6.98 $/kg), wind-driven (8.01 $/kg), and solar-based (16.01 $/kg) hydrogen production [45]. Although fossil fuel-based hydrogen production is more economical than environmentally friendly hydrogen production, most unsustainable technologies heavily emit CO_2,_ causing potential global warming problems. Hence, renewable hydrogen sources such as solar, wind, geothermal, biomass, and nuclear-assisted electrolysis could prove to be promising and comparative alternatives, offering a sustainable means of catalytic BrO_3_^−^ removal [46,47,48]. It is possible for the transition to clean hydrogen to replace the use of conventional environmental technologies with novel catalytic systems in order to meet the most recent sustainable environmental standard goals in water and wastewater treatment. 

Catalyst durability is also a vital factor and essential to consider for its actual implementation. The catalytic performance of the nanoscale heterogeneous catalysts used for BrO_3_^−^ reduction has been widely demonstrated to decline continuously over repeated cycles of the contaminated water and groundwater treatment in batch and continuous reactors. However, the longevity of the nanocatalysts is significantly improved during the constant supply of H_2_, preventing the decrease in catalytic stability and reactivity mainly attributed to the oxidation of the support materials and/or active catalyst sites [12]. An enhancement of catalyst durability has the potential to significantly reduce the cost-related limitations of the technology. 

## 5. Concluding Remarks and Perspectives 

The process of catalytic BrO_3_^−^ reduction by nanoscale heterogeneous catalysts with H_2_ is a reliable and practical environmental technology for water and groundwater treatment. The catalytic behavior of nanoscale heterogenous catalysts in different environmental system configurations with and without gaseous H_2_ and solid-state H_2_ sources has been comparatively evaluated and assessed. The implementation of H_2_ gas use in the catalytic system still has disadvantages and concerns in its application to the actual large-scale treatment processes that need to be overcome. Through the proper acquisition of H_2_ sources, the limitations in storing, transportation, cost-effectiveness, and sustainable production can be managed and overcome to successfully implement catalytic BrO_3_^^−^^ reduction in actual water and wastewater treatment. Future research needs to focus on an in-depth investigation of the usable storage and transportation of H_2_ specifically designed for water and groundwater treatment. Novel environmental materials with suitable properties that provide for the complete and efficient consumption of H_2_ during BrO_3_^^−^^ reduction and minimize safety issues also need to be developed and examined in order to attract investments in pilot-scale studies. 

## Figures and Tables

**Table 1:** The reactivity of different nanoscale heterogeneous catalysts for the reduction of aqueous BrO_3_^−^.

Catalyst	Bromate Concentration (mg·L^−1^)	Catalyst Dose (mg∙L^−1^)	Source of Hydrogen, Hydrogen Flow (mL (STP) min^−1^)	Reduction Efficiency (Time)	Bromide Generation (%)	Effective pH Range	References
Ru/C, Pt/C and Pd/C	10	500	No	100% (120 min)	~100	3–5	[13]
G-NZVI	50	200	No	100% (2 min)	100	7	[16]
NZVI/MAC	0.2	5	No	100% (5 min)	83.1	3–8	[15]
NZVI (Cu-Pd)	25	50	H_2_ gas, 40	>99%, (11 h)	100	-	[12]
Metal (Pd, Ru) CNF/monolith catalysts	50	200	H_2_ gas, 250	~70% (<25 min)	~95	-	[17]
Pd, Rh, Ru and Pt supported on activated carbon	10	400	H_2_ gas, 100	100%, (<30 min)	100	-	[18]
Pd/Cu-Y (metals over faujasite zeolite)	10	150	H_2_ gas, 50	100% (2 min)	~100	-	[19]
Pd/mesoporous carbon nitride	100	30	H_2_ gas, 40	100% (50 min)	~100	2–5.6	[20]
Mono and bimetallic (Cu-Pd) ZSM5	10	500	H_2_ gas, 50	100% (10 min)	100	-	[21]
Ni(4,4′-bipy)(1,3,5-BTC)	25	500	NaBH_4_	100% (15 min)	>95	3–7	[22]
ZIF-67 (carbonized)	100	500	NaBH_4_	100% (60 min)	100	3–10	[23]
MIL-88A	100	500	NaBH_4_	100% (60 min)	100	3–5	[24]
ZIF-67	100	500	NaBH_4_	100% (60 min)	100	3–5	[24]

**Table 2:** A summary of durability test results by nanoscale heterogeneous catalysts for repeated cycles of BrO_3_^−^ reduction.

Catalyst	Number of Cycles	Removal Efficiency	Metal Leaching	Reference
Ru/C, Pt/C and Pd/C	>70% after 5th cycle	80%	-	[13]
nZVI/MAC	-	100%	No metal leaching	[15]
NZVI (Cu-Pd)	24 h continuous	>99%	Negligible amounts of leaching for Fe, Cu, Pd	[12]
Metal (Pd, Ru) CNF/monolith catalysts	10% loss, 7 h continuous	~70%	No metal leaching	[17]
Pd, Rh, Ru and Pt supported on activated carbon	5	100%	-	[18]
Pd/Cu-Y (metals over faujasite zeolite)	2	100%	No metal leaching	[19]
Pd/mesoporous carbon nitride	-	100%	-	[20]
Mono and bimetallic (Cu-Pd) ZSM5	3	100%	Cu, less than 1% of the initial amount; negligible	[21]
(Ni(4,4′-bipy)(1,3,5-BTC)	6	100%	Negligible, 0.002 µg⋅L^−1^	[22]
ZIF-67 (carbonized)	4	100%	-	[23]
MIL-88A	5 (with minor loss)	100%	No metal leaching	[24]
ZIF-67	5	100%	No metal leaching	[24]

## Data Availability

Data sharing is not applicable to this article as no new data were created or analysed in this study.
